# The Criminal Boy

**Published:** 1916-06

**Authors:** 


**The Criminal Boy.** Wm. O. Krohn, Chicago, Ill., Med. Jour., May. 112 cases were investigated, mostly inmates of the Cook Co. Jail. 9.5% had defective vision, mostly minor astigmatism, average for Illinois school children 11%. 18% had defective hearing mostly from adenoids, sequelae of scarlatina and catarrhal disease, average for school children 19%. The criminal boys showed a notable increase in perversions of taste but mostly on account of the local effects of cigarette smoking. Touch was somewhat blunter than among average children. Visual was better than auditory memory, as for most persons and concrete examples were better solved than abstract. For example, the boys could add well enough in playing rhum but had difficulty with identical numbers in the abstract. Judgment, imagination and reasoning showed shrewdness and practical intelligence but a failure to comprehend fine distinctions, especially of a moral nature. The author very sensibly protests against the tendency to excuse delinquency solely on account of age and thinks that lenity for first detected crimes tends to confirm the criminal in his practices.

